# Tumor-Associated Regulatory T Cell Expression of LAIR2 Is Prognostic in Lung Adenocarcinoma

**DOI:** 10.3390/cancers14010205

**Published:** 2021-12-31

**Authors:** Dalam Ly, Quan Li, Roya Navab, Cédric Zeltz, Linan Fang, Michael Cabanero, Chang-Qi Zhu, Ming-Sound Tsao, Li Zhang

**Affiliations:** 1 Toronto General Hospital Research Institute, University Health Network, Toronto, ON M5G 1L7, Canada; dalam.ly@uhnresearch.ca (D.L.); fanglinan_jdyy@jlu.edu.cn (L.F.); 2 Department of Immunology, University of Toronto, Toronto, ON M5G 1X8, Canada; 3 Princess Margaret Cancer Centre, University Health Network, Toronto, ON M5G 1L7, Canada; quan.li@uhnresearch.ca (Q.L.); Roya.Navab@uhnresearch.ca (R.N.); cedric.zeltz@wanadoo.fr (C.Z.); michael.cabanero@uhn.ca (M.C.); integrina11@gmail.com (C.-Q.Z.); 4 Department of Laboratory Medicine and Pathobiology, University of Toronto, Toronto, ON M5G 1X8, Canada

**Keywords:** *LAIR2*, T_reg_ cells, collagen receptor, prognostic gene, human lung carcinoma

## Abstract

**Simple Summary:**

Understanding how the immune system navigate the tumor microenvironment is vital to developing effective drugs to treat cancer. Using gene and functional studies, we found that the collagen receptor LAIR2 is an important component of cancer regulation. When expressed in regulatory T cells, a LAIR2 containing gene signature is adversely prognostic in lung cancer. This study highlights the importance of microenvironment regulation of immune cells and provides a unique target for future therapeutic development.

**Abstract:**

Cancer development requires a permissive microenvironment that is shaped by interactions between tumor cells, stroma, and the surrounding matrix. As collagen receptors, the leukocyte-associated immunoglobulin-like receptor (LAIR) family allows the immune system to interact with the extracellular matrix. However, little is known about their role in regulating tumor immunity and cancer progression. Methods: Genetic analysis of resected human lung adenocarcinoma was correlated to clinical-pathological characteristics, gene ontologies, and single cell RNA sequencing (scRNASeq). LAIR2 production was determined in subsets of immune cells isolated from blood leukocytes and lung adenocarcinoma tumor. Functional assays were used to determine the role of LAIR2 in tumorigenesis. Results: *LAIR2* expression was adversely prognostic in lung adenocarcinoma. LAIR2 was preferentially produced by activated CD4^+^ T cells and enhanced in vitro tumor invasion into collagen. scRNASeq analysis of tumor infiltrating T cells revealed that *LAIR2* expression co-localized with FOXP3 expressing cells and shared a transcriptional signature with tumor-associated regulatory T (T_reg_) cells. A CD4^+^ LAIR2^+^ T_reg_ gene signature was prognostically significant in the TCGA dataset (*n* = 439; hazard ratio (HR) = 1.37; 95% confidence interval (CI), 1.05–1.77, *p* = 0.018) and validated in NCI Director’s Challenge lung adenocarcinoma dataset (*n* = 488; HR = 1.54; 95% CI, 1.14–2.09, *p* = 0.0045). Conclusions: Our data support a role for LAIR2 in lung adenocarcinoma tumorigenesis and identify a CD4^+^ LAIR2^+^ T_reg_ gene signature in lung adenocarcinoma prognosis. LAIR2 provides a novel target for development of immunotherapies.

## 1. Introduction

Lung cancer is the leading cause of cancer-related deaths world-wide, and 85% of patients belong to non-small cell lung cancer (NSCLC), of which lung adenocarcinoma (LUAD) and lung squamous cell carcinoma (LUSC) are the most common subtypes [1]. Immunotherapies targeting immune checkpoint PD-1/PD-L1 interaction have now become standard of care in the treatment of advanced stage NSCLC patients. However, only subsets of patients respond. Even among patients who have high expression of PD-L1, more than 50% of patients do not respond [1,2,3]. This may be partially due to our incomplete understanding of the tumor microenvironment (TME) and its relation to lung cancer biology [4,5].

TME has a significant role in shaping cancer development, tumor progression and patient prognosis. Cancer growth requires the presence of a permissive TME that is shaped by interactions between tumor cells and the stroma, which consist of immune cells, fibroblasts, endothelial cells, and stromal matrices [6,7]. Several groups have described tumor stromal immune signatures and histologically defined immune cell subsets associated with NSCLC prognosis [8,9,10]. CD4^+^ regulatory T (T_reg_) cells that express the transcription factor Foxp3 are an important T cell subset that are recruited to sites of inflammation and facilitate tumor progression and metastasis in most cancers [11,12,13]. Though T_reg_ cells have been associated with unfavorable prognosis in NSCLC, meta-analysis suggests that T_reg_ cells represent a highly heterogeneous population which are shaped by their microenvironment [14,15,16]. A growing body of evidence indicates that tumor-associated T_reg_ cells have unique transcriptional signatures relative to their peripheral blood counterparts [17,18,19,20,21,22].

Amongst immune expressed genes, the leukocyte-associated immunoglobulin-like receptor (LAIR) family allow the immune system to interact with the extracellular matrix, due to its ability to bind collagen and collagen-motif containing proteins [23,24,25,26]. The LAIR family comprise of two members, LAIR1 and LAIR2. LAIR1 (CD305) is a known receptor for collagen type I with inhibitory activity on multiple types of immune cells [27,28,29]. In contrast, LAIR2 (CD306), which shares ~84% homology to LAIR1, lacks transmembrane and signaling domains and is a secreted receptor that could serve as a competitive inhibitor of LAIR1 [24,25]. LAIR2 is elevated in the serum and synovial fluid of patients with rheumatoid arthritis [25,30] and in the serum of patients with autoimmune thyroid disease [31], suggesting a role for LAIR2 at sites of inflammation. In addition, LAIR2 has been implicated in rescuing CD8^+^ T cells response towards anti-PD-1 therapy in murine models of cancer [23]. However, the role of LAIR2 in human cancer progression and immunity is unknown. In this study, we demonstrated a prognostic role for tumor infiltrating CD4^+^ LAIR2^+^ T_reg_ cells in lung adenocarcinoma.

## 2. Materials and Methods

### 2.1. Patient and Gene Expression Data Analyses

Microarray gene expression data of 181 stage I–II NSCLC patients (UHN181) (GSE50081) were analyzed for potentially prognostic immune-related genes [32]. The immune-related genes were those in the National Institute of Allergy and Infectious Diseases (NIAID) gene list (ImmPort Comprehensive List of Immune-Related Genes, version 2011_08_01). Cox proportional regression model was used to assess their association with 5 year survival and identified LAIR2 as prognostically significant.

### 2.2. Pathway Enrichment, Gene Set Enrichment Analysis and Immune Estimation

To determine biological pathways associated with *LAIR2* expression, ClueGO and CluePedia applications were used within network visualization platform Cytoscape, as previously described [33,34]. The top 500 JetSet v1.6 selected probes from correlating with *LAIR2* expression (Pearson’s correlation co-efficient r > 0.37; *p* < 0.00001) were used for Gene Ontology (GO) analysis within ClueGO. GO analysis was performed using the GO BiologicalProcess-EBI version 20.11.2017 dataset. Terms found in the 3–8 GO interval, with at least 3 genes from the initial list representing minimum 4% were selected with a kappa score of 0.68, with *p* < 0.05 significance. GO Fusion was applied to reduce the redundancy. Gene set enrichment analysis (GSEA) [35] was performed using the javaGSEA with a priori defined gene signatures [36]. Gene signatures were mapped to Affymetrix HG_U133_Plus2 microarray probes and significantly enriched signatures were determined using a Pearson metric after generating a continuous phenotype using *LAIR2* (207509_s_at) expression within the University Health Network (UHN) dataset. Immune deconvolution analysis xCell [37] was used to estimate immune cell subset enrichment scores within the top and bottom quartile of *LAIR2* expressing patients with UHN LUAD and enrichment scores were correlated to *LAIR2* expression.

### 2.3. Cell Isolation, Expansion and ELISA

Peripheral blood mononuclear cells (PBMCs) from healthy donors and tumor-infiltrating lymphocytes from LUAD patients were obtained with consent and a protocol approved by the UHN Research Ethics Board (#05–0221). For selection of CD4^+^, CD8^+^, CD3^−^CD56^+^ natural killer (NK), and CD3^−^CD56^−^ cells, PBMCs were sequentially enriched for each subset by first selecting for NK cells with CD56 microbeads. To select for CD8^+^ T cells, CD8 microbeads were used against the CD56-negative flow through cells. To select for CD4^+^ T cells, CD4 microbeads were used against the CD56-CD8-negative flow through cells, with the remaining flow through collected as the CD3^−^CD56^−^ negative fraction. Enriched populations were each cultured at 1 × 10^6^ cells/mL in the presence of 1× phorbol myristate acetate (PMA)/ionomycin (eBioscience Cell Stimulation Cocktail (500×), ThermoFisher, Waltham, MA, USA) for 4 d and supernatants collected for ELISA analysis [25]. To expand tumor-infiltrating T cells (TILs), fresh lung resection was dissociated in Liberase solution (0.5 mg/mL, Sigma Aldrich, St. Louis, MO, USA) for 45 min at 37 °C, followed by treatment for 5 min with red blood cell lysis buffer (0.15 M NH_4_Cl, 0.01 M KHCO_3_, 0.1 mM Na_2_EDTA). The resulting cell suspension was filtered through a 70 µm filter to remove debris. Dissociated cells were plated overnight with IL-2 (2000 U/mL) and on day 3, expanded with irradiated K562 artificial antigen-presenting cells (aAPC) expressing mOKT3, CD86, and 41BBL (kindly provided by Dr. Naoto Hirano, Princess Margaret Cancer Centre, UHN, Toronto, Canada) at 2:1 aAPC:T cell ratio in the presence of IL-2 (100 U/mL), IL-15 (10 ng/mL), and IL-21 (10 ng/mL). Expanding TILs were restimulated with aAPC and cytokines every 4–7 d. After 15 d of expansion, TILs were sequentially selected for CD8, with negative flow through selected for CD4 using positive selection microbeads and 1 × 10^6^ cells/mL were restimulated in the presence of 1× PMA/ionomycin (eBioscience Cell Stimulation Cocktail) for 4 d and supernatants collected for analysis. LAIR2 was detected in supernatants from various cell cultures using LAIR2 ELISA kit (SinoBiological US Inc., Wayne, PA, USA).

### 2.4. Cell Adhesion and Invasion Assay

NCI-H661, HCC4006, and HCC827 lung cancer cell lines were obtained from the American Type Culture Collection (ATCC) and were cultured in Roswell Park Memorial Institute (RPMI) 1640 media (ThermoFisher, catalogue: 11875093) supplemented with 10% fetal bovine serum (FBS) (Sigma Aldrich, catalogue: F1051). To determine adhesion of cell lines towards immobilized protein, 96-well plates were incubated with 2% BSA, 10 µg/mL rat collagen I or 10 µg/mL rhLAIR2 for 1 h at 37 °C (R & D Systems, Minneapolis, MN, USA). The next day, plates were washed and blocked with 2% BSA prior to addition of tumor cells. Tumor cells were trypsinized and washed twice with RPMI. The 10^5^ cells/well were seeded on the plate and incubated for 40 min at 37 °C. Following incubation, non-adherent cells were washed away and cell adhesion was quantified using crystal violet, absorbance 595 nm. To inhibit collagen mediated binding, rhLAIR2 (10 µg/mL) was added to cell culture media during 40 min period. For 3D migration assays, tumor cells were cultured as homospheroid using the Nunclon Sphera plate (VWR, Mississauga, ON, USA) in co-culture with HuT78 T cells (ATCC) previously transduced using lentivirus to express GFP or LAIR2 (GeneCopoeia, Rockville, MD, USA). Briefly, confluent monolayers were trypsinized, resuspended as single cells in RPMI + 10% FBS and 8000 cells were plated in drop of 40 µL per well of 96-well Sphera-plate. After 48 h, spheroids were flushed in 160 µL/well of collagen solution comprising of 50,000 Hut78 T cells suspended in 9 volumes of rat tail collagen I (3.8 mg/mL; Advanced BioMAtrix; San Diego, CA, USA), 1 volume of neutralization solution (Advanced BioMatrix), and 8.5 volumes of 2× RPMI medium. After 10 d, the 3D-matrix invasion areas were analyzed using texture analysis available as a plugin for the freeware ImageJ analysis software (http://imagej.nih.gov/ij/index.html, accessed on 16 April 2019) to evaluate the pixels in different directions around the spheroid, which is a measure of invading area. All images were taken on an EVOS 700 cell imaging system (ThermoFisher).

### 2.5. Single Cell RNASeq Analysis

Single cell RNASeq dataset GSE99254 was obtained from Gene Expression Omnibus (GEO) and normalized to log_2_ transcripts per kilobase million (TPM) + 1. 2D analysis of individual features extracted from datasets was generated and displayed as density plots in FlowJo v10. t-distributed stochastic neighbor embedding (t-SNE) plots were generated from online resource of dataset GSE99254 available at (http://lung.cancer-pku.cn/, accessed on 3 March 2019). To generate the CD4^+^ LAIR2^+^ T_reg_ gene signature, a *t*-test was used on the GSE99254 scRNASeq dataset to identify genes that are differentially expressed between CD4^+^ LAIR2^+^ and CD4^+^ LAIR2^−^ T cell clusters. We identified 231 genes highly expressed in the CD4^+^ LAIR2^+^ cluster (Benjamini–Hochberg-adjusted *p*-value < 0.001 and fold change ≥ 1.55). This set of 231 genes were defined as CD4^+^ LAIR2^+^ high TILs signature. The LAIR2 signature scores were calculated as the average of the across-sample normalized log_2_ median-centered expression levels of these 231 signature genes. The samples in each cohort were split into high- and low-survival risk groups based on LAIR2 signature score using median as the cut-off. The CD4^+^ LAIR2^+^ signature was validated using LUAD specimens from the TCGA Research Network (https://www.cancer.gov/tcga, accessed on 10 March 2020) and NCI Director’s Challenge lung adenocarcinoma dataset (ca00182) [38] of resected tumors with available clinical and survival data.

### 2.6. Statistical Analysis

The R package survival and survminer were used for the survival analysis. The primary endpoint was overall survival (OS), and survival time was measured from the date of diagnosis until the date of death or date last seen alive. Kaplan–Meier plots with log-rank test to determine significance of OS and Cox proportional hazards regression to determine relative risk of death were used to analyze the survival data. The R package metaphor [39] was used to calculate the overall HR.

## 3. Results

### 3.1. High LAIR2 Expression Is Associated with Poor LUAD Patient Survival

**Figure 1:**
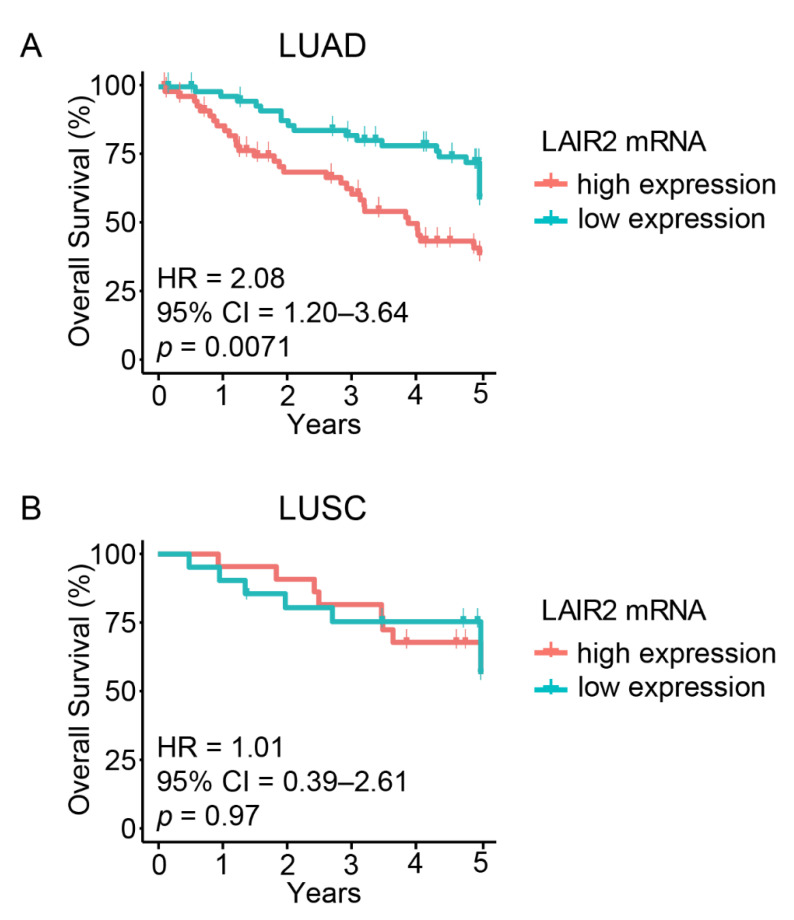
*LAIR2* expression is associated with negative prognosis in LUAD. (**A**), Univariate Kaplan–Meier plots showing OS of LUAD patients or (**B**), LUSC patients within the UHN181.

To identify immune-related genes that are significantly prognostic, we separately explored the gene expression profiles of LUAD (*n* = 128) and lung squamous cell carcinoma (LUSC, *n* = 53) patients [32]. High *LAIR2* expression was univariately associated with poorer OS (Figure 1A; HR = 2.08, 95% CI 1.20–3.64, *p* = 0.0071) in LUAD, but was not prognostic among LUSC patients (Figure 1B; HR = 1.01, 95% CI 0.39–2.61; *p* = 0.97). Furthermore, *LAIR1,* the most closely related gene family member of *LAIR2*, was also not prognostic (Figure S1). *LAIR2* expression was independent of known pathologic-clinical correlations including age, sex, smoking history, histological pattern or stage (Table S1) and after adjusting for these factors, multivariable analyses showed that *LAIR2* expression and stage remained significantly prognostic (Table 1).

### 3.2. CD4^+^ T Cells Are a Major Source of Soluble LAIR2

**Figure 2:**
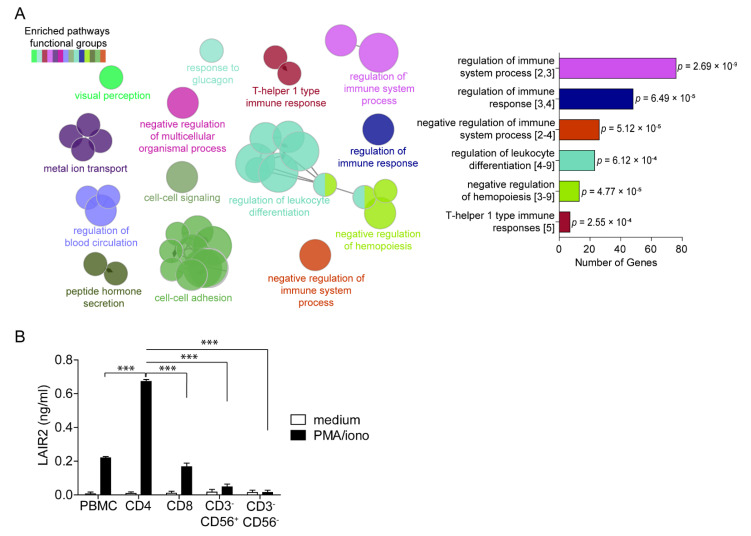
*LAIR2* expression is associated with immune cells and secreted by CD4^+^ T cells. (**A**), Left, ontological networks formed using ClueGO/CluePedia from genes co-expressed with *LAIR2* (Table S2). Right, bar graph shows number of genes and significance of GO. Square brackets denote GO level hierarchy with terms on the lower levels (greater number) being more specific, while terms on higher levels being more general in their biological definition. (**B**), LAIR2 secretion by sorted PBMCs stimulated with PMA/ionomycin for 4 d or left unstimulated, data are representative of two healthy donors. *** *p* < 0.0001 as indicated, determined by two-way ANOVA test.

To understand the role of LAIR2 in lung cancer, we performed GO analysis, querying biological processes within our bulk transcriptomic dataset and found that the most significant gene ontologies associated with *LAIR2* expression were involved in immune-related functions. The largest network of genes concerns the regulation of immune system processes (76 genes, *p* = 2.69 × 10^−9^) (Figure 2A), suggesting that *LAIR2* expression was associated with immune, rather than epithelial, compartment regulation. Furthermore, gene set enrichment analysis (GSEA) using curated gene sets with biologically defined functions in lung cancer immunity [36] showed that a high expression of *LAIR2* was associated with “T cell signatures of lung cancer” and “exhaustion”, but not resident memory T cells (Figure S2). These data suggest that *LAIR2* expression may regulate tumor infiltrating populations rather than tissue resident cells.

To determine which subset of immune cells might secrete LAIR2, purified CD4^+^, CD8^+^, CD3^−^CD56^+^ NK, and CD3^−^CD56^−^ cells, together with unsorted healthy donor, PBMCs were stimulated in vitro and their production of LAIR2 was measured. We found that NK cells and CD3^−^CD56^−^ cells produced minimal level of LAIR2 whereas CD8^+^ T cells and unsorted PBMC secreted low levels of LAIR2. Interestingly, the predominant source of LAIR2 was derived from CD4^+^ T cells where CD4^+^ T cells produced a 4-fold greater amount of LAIR2 than CD8^+^ T cells (*p* < 0.0001, Figure 2B). Collectively, these data indicate that *LAIR2* expression was associated with immune processes and that LAIR2 was predominantly secreted by CD4^+^ T cells.

### 3.3. T Cell Derived LAIR2 Enhances Tumor Cell Invasion

**Figure 3:**
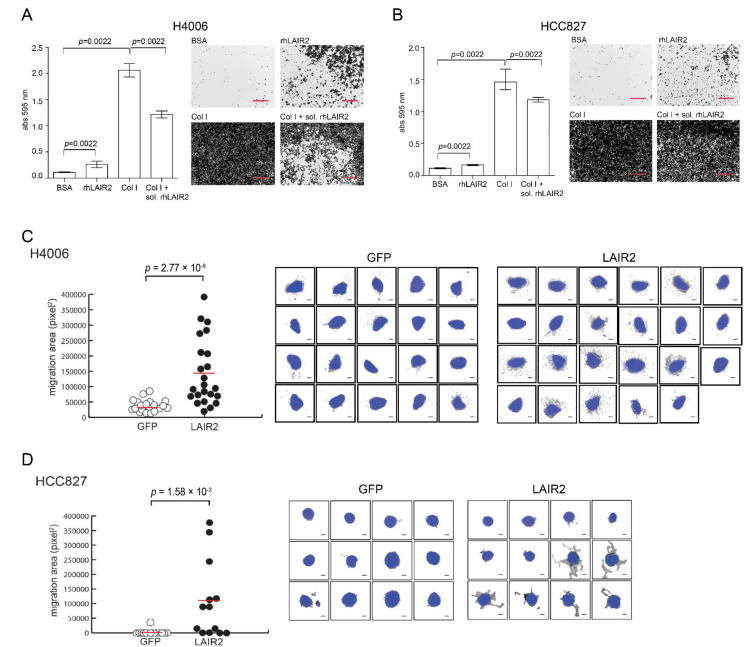
LAIR2 expression enhances NSCLC tumor invasion into collagen matrix. (**A**,**B**), HCC4006 and HCC827 NSCLC adherence was measured towards indicated immobilized proteins, or in the presence of soluble rhLAIR2. Non-adherent cells were removed after 40 min and adherent cells were quantified using crystal violet absorption and measured by spectrophotometry. Images taken at 4× magnification. Red scale bars = 500 µm (**C**,**D**), HCC4006 and HCC827 tumor invasion into collagen was measured in the presence of paracrine LAIR2. HCC4006 and HCC827 spheroids were embedded in type 1 collagen containing T cell line Hut78 transduced to express LAIR2 or mock (GFP) vector. Migration area from initial spheroid (in blue) was determined after 8 d of co-culture using texture analysis. Black scale bars = 200 µm. Data are representative of two independent experiments. *p*-values were determined by Mann–Whitney test.

With the finding that T cells were a source of LAIR2 secretion, we sought to determine if LAIR2 may have an effect on tumor cell adhesion and invasion. Using cellular adhesion assays, we first found that recombinant human LAIR2 (rhLAIR2), immobilized onto a plate, was capable of increasing cellular adhesion of human NSCLC cell lines HCC4006 and HCC827, relative to the BSA negative controls (Figure 3A,B). Additionally, we observed that the addition of soluble rhLAIR2 significantly reduced the adherence of NSCLC cell lines HCC4006 and HCC827 (*p* = 0.0022) towards plate-bound collagen I (Figure 3A,B), collectively suggesting that LAIR2 and collagen I may interact with similar receptors on tumor cells.

With rhLAIR2 having the ability to alter cancer cell binding to collagen, we modeled its presence within the TME by overexpressing LAIR2 in Hut78 T cells and determined if paracrine expression may alter tumor invasion into extracellular matrix. HCC4006 and HCC827 spheroids were embedded in rat type I collagen matrix (the type of collagen that can get cross-linked) containing T cells secreting LAIR2. Consistent with the oncogenic role of LAIR2, matrix embedded with T cells secreting LAIR2, but not control (GFP), were capable of increasing invasion of both HCC4006 and HCC827 into matrix. This led to ~3- or 10-fold increase in the number of invasive foci entering matrix, respectively (Figure 3C,D), indicating that LAIR2-producing T cells may alter tumor cell migration.

### 3.4. LAIR2 Is Expressed by Tumor-Associated T_reg_ Cells

**Figure 4:**
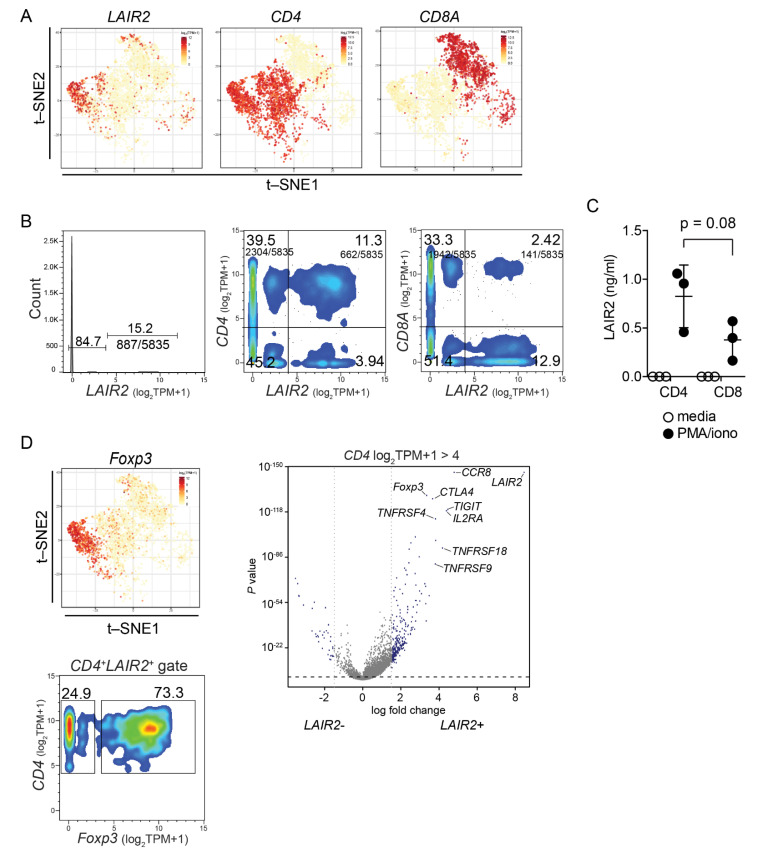
LAIR2 is a marker of tumor-associated Foxp3^+^ T_reg_ cells. (**A**), t-SNE plots of *LAIR2* and T cell co-receptors from scRNASeq dataset of NSCLC patient tumors. (**B**), scRNASeq dataset displayed as histogram of *LAIR2* expression and as pseudocolor density plots of individual cells with frequency and number of events for indicated genes within quadrants and gates. (**C**), LAIR2 secretion by expanded TILs stimulated with PMA/ionomycin for 4 d. Dots represent individual patient TILs expansions. *p*-value was determined using one-way ANOVA with Turkey’s post-test. (**D**), t-SNE plot of *Foxp3* and volcano plot of genes expressed by *CD4^+^* T cells with high or low expression of *LAIR2* at cut-off of ≥4 log_2_ (TPM + 1) expression. Displayed are t-SNE dimensions 1 and 2, with each data point representing a single cell, colored by the intensity of expression log_2_ (TPM + 1) of indicated gene. scRNASeq displayed as pseudocolor density plot of individual cells with frequency of events for indicated gates.

Though peripheral T cells, particularly CD4^+^ T cells can secrete LAIR2, it is important to know the origin of *LAIR2* within tumor tissue T cells. We took advantage of single cell RNASeq (scRNASeq) dataset of TILs derived from 14 surgically resected treatment naïve NSCLC patients that contained expression data from ~9000 sorted CD4^+^ and CD8^+^ T cells derived from TILs [17]. Consistent with observed biased secretion of LAIR2 by peripheral CD4^+^ T cells, the majority of *LAIR2* expressing TILs co-localized with CD4^+^ T cell expression (Figure 4A). Further, *LAIR2* expression could be defined within T cells as either being *LAIR2^+^* or *LAIR2^−^* at a defined cut-off of log_2_(TPM + 1) > 4 or <4. When segregating cells this way, 11.3% of all TILs co-expressed *LAIR2* and *CD4*, while only 2.4% of TILs co-expressed *LAIR2* and *CD8A* at log_2_(TPM + 1) > 4 (Figure 4B), indicating differences in *LAIR2*^+^ expression between different T cell compartments of the tumor samples. This was in contrast to *LAIR1* expression, which was found to be distributed amongst all clusters of T cells (Figure S3A), consistent with its known ubiquitous distribution [24]. To confirm that CD4+ T cells were the major source of LAIR2 in TILs, we expanded three NSCLC patient TILs samples and analyzed LAIR2 secretion. Though not reaching statistical significance, we observed a trend consistent with biased expression of LAIR2 as seen in scRNASeq data and found a 2-fold higher secretion of LAIR2 by tumor infiltrating CD4^+^ T cells than CD8^+^ T cells (Figure 4C).

Since the majority of *LAIR2*^+^ cells within TILs derived from CD4^+^ T cells, we queried the scRNASeq dataset by comparing the expression profile of *CD4*^+^ *LAIR2*^+^ cells with that of *CD4*^+^ *LAIR2*^−^ cells within the tumor-infiltrating fraction. Analyzing this way we found that *CD4*^+^ *LAIR2*^+^ cells highly expressed the T_reg_ cell lineage marker *FOXP3* with t-SNE plots showing co-localization of *FOXP3* and *LAIR2* expression (Figure 4D). By gating specifically on *CD4*^+^ *LAIR2*^+^ TILs, we found that more than 70% of cells expressed the T_reg_ transcription factor *FOXP3* at log_2_ (TPM + 1) > 4 (Figure 4D, lower panel). *CD4*^+^ *LAIR2*^+^ cells highly expressed T_reg_ cell lineage markers, including *IL2RA, CTLA4, TNFRSF18, ICOS*, *TIGIT,* and tumor-associated T_reg_ cell marker *CCR8* [18] (Figure 4D, right panel; Figure S3B), consistent with *CD4^+^ LAIR2^+^* cells being of T_reg_ lineage and not recently activated T cells. Additionally, as dropout events are known to occur in scRNASeq platforms, we confirmed the association of LAIR2 with T_reg_ cell gene signature within bulk genomic dataset using gene expression deconvolution algorithm xCell (Figure S4A). We found that *LAIR2* expression was negatively associated with mast, endothelial and epithelial cell enrichment scores (Spearman’s rank correlation r_s_ = −0.402; *p* = 0.001), but positively associated with T_reg_ cell enrichment score (r_s_ = 0.482; *p* < 1.00 × 10^−5^) (Figure S4B). Collectively, our results support the notion that LAIR2 expression marks the presence of tumor-associated T_reg_ cells.

### 3.5. CD4^+^ LAIR2^+^ Tumor-Associated T_reg_ Gene Signature Is Prognostic in LUAD

**Figure 5:**
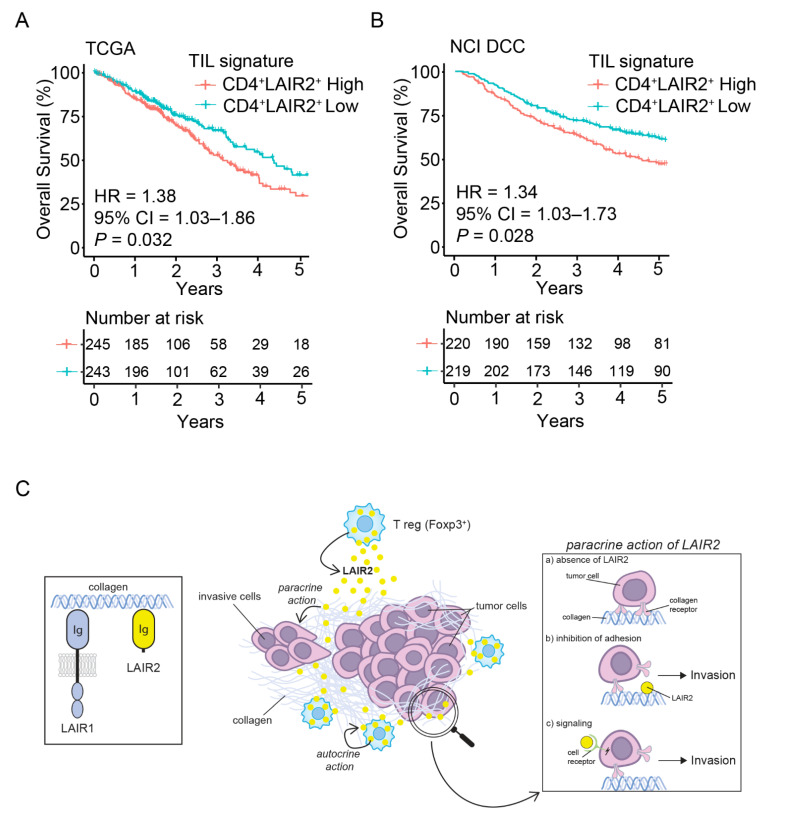
Tumor-associated CD4^+^ LAIR2^+^ gene signature is prognostic in NSCLC validation. Kaplan–Meier plots showing OS of LUAD patients in (**A**), TCGA, (**B**), NCI Director’s Challenge Consortium datasets, patients were divided into two risk groups based on the median value of the gene signature risk scores. Number at risk indicates the number of participants who are event free at indicated times. (**C**), Schematic of LAIR2 activity. LAIR2 is a secreted protein that interacts with collagen through its Ig domain. In contrast to LAIR1, LAIR2 lacks transmembrane and signaling domains. Amongst tumor infiltrating T cells, LAIR2 was found to be predominately expressed by tumor-associated Foxp3^+^ Treg cells by scRNASeq. As a ligand for collagen, autocrine LAIR2 may help Treg cells function in a collagen dense environment. In addition, LAIR2 was found to act in a paracrine fashion by increasing tumor migration and invasion into collagen matrix. As a mechanism, LAIR2 inhibits tumor cell adhesion on collagen that could initiate tumor migration and invasion. Along with inhibition of adhesion, interaction of LAIR2 with a cell receptor that mediates signaling leading to cell invasion cannot be excluded.

Having initially found *LAIR2* to be adversely prognostic, we sought to validate the prognostic association of *LAIR2* in additional LUAD patient cohorts. Using TCGA (*n* = 439) and the NCI Director’s Challenge Consortium (DCC) LUAD (*n* = 488) datasets, we found that the gene signature associated with *CD4*^+^ *LAIR2*^+^ TILs (Figure 4D and Table S3) was significantly prognostic in both TCGA (HR = 1.38; *p* = 0.032, Figure 5A) and DCC (HR = 1.34; *p* = 0.028, Figure 5B) datasets and was predictive of adverse patient outcomes, validating our initial discovery. Notably, the *CD4*^+^ *LAIR2*^+^ TILs cell signature is significant across both RNA sequencing (TCGA) and microarray (DCC) datasets. Additionally, when the presence of CD4^+^ T cells was taken into consideration, the *CD4*^+^ *LAIR2*^+^ TILs signature gained greater significance within each dataset, TCGA (HR = 1.54; *p* = 0.0045, Figure S5A) and DCC (HR = 1.37; *p* = 0.018, Figure S5C), which was not observed when normalized to CD8^+^ T cells (Figure S5B,D), confirming the association between LAIR2 expression and presence of CD4^+^ tumor-associated T_reg_ cells. Overall, these results demonstrate that *LAIR2* expression may define subsets of adversely prognostic Foxp3 T_reg_ cells.

## 4. Discussion

Using patient derived datasets, we found that the presence of the soluble collagen receptor *LAIR2* was associated with poorer prognosis in early stage LUAD patients. *LAIR2* expression was correlated with gene ontologies involving negative immune regulations. From single-cell transcriptomics of TILs, *LAIR2* was found to be predominantly expressed by tumor-associated CD4 T_reg_ cells, to which a LAIR2-T_reg_ cell derived signature was adversely prognostic in LUAD. In addition, we found that T cell derived LAIR2 was capable of increasing tumor cell invasion into extracellular collagen matrix.

Amongst tumor microenvironmental factors, receptors that interact with extracellular matrix components, such as collagen, play important roles in regulating tumor progression. Alterations to the biophysical property of collagen, such as the stiffening of collagen within the TME can lead to increased tumor migration and metastasis [40,41,42,43,44]. The LAIR family of proteins are immune expressed collagen receptors that link the extracellular matrix to the immune system [24]. LAIR1, an inhibitory receptor expressed basally on the majority of immune cells has been shown to bind collagen and inhibit CD8^+^ T cells [23,24]. In contrast, LAIR2 which lacks a transmembrane domain and is known to be secreted, has been postulated to compete with LAIR1 and thus alleviate collagen-induced immune inhibition [25]. Indeed, in a murine model of lung cancer, the overexpression of LAIR2 within tumor cells was found to rescue collagen-induced inhibition of anti-tumor CD8^+^ T cells and response to anti-PD1 therapy, resulting in tumor reduction [23]. Paradoxically, from our transcriptomic analysis of human LUAD, we found that *LAIR2* expression was associated with adverse patient outcomes and signatures of negative immune regulation rather than conditions associated with favorable tumor immunity. Interestingly, this was not observed in our LUSC discovery dataset, which shows no statistical role for LAIR2 in prognosis (Figure 1B). This is likely due to differences in the immunologic signatures observed between LUAD and LUSC, which influence the composition of immune infiltrates that contribute to prognosis [10,45,46]. Whereas LUAD prognosis is strongly associated with the presence of B cells, LUSC prognosis is associated with a lack of myeloid dendritic cells and neutrophil signatures, highlighting major differences in the immune regulation of theses NSCLC subtypes [46].

Although experimental models provide evidence that LAIR2 can abrogate collagen-induced LAIR1-mediated inhibition, given the ubiquitous presence of LAIR1 on most immune cells, it remained unclear which cells may be regulated from the presence of LAIR2 in situ. We show that CD4^+^ T cells derived by PBMCs or TILs were the major producers of LAIR2 (Figure 2B), highlighting differences observed between patient tissue and experimental systems [23]. Using scRNASeq of TILs from LUAD, we found that *LAIR2* was predominantly expressed by tumor-associated CD4^+^ FoxP3^+^ T_reg_ cells (Figure 4), the presence of which have widely been associated with negative outcomes in a variety of cancers [11,12,13]. Though we found ex vivo expanded CD4^+^ and CD8^+^ T cells derived from PBMCs or TILs capable of LAIR2 secretion (Figure 2B and Figure 4C), selective expression by tumor-associated T_reg_ cells in situ suggests that the tumor microenvironment may have a role in regulating *LAIR2* expression. Although *LAIR2* was found to be associated with global signatures of T cell exhaustion (Figure S2), we show for the first time that *LAIR2* was co-expressed with markers found on highly activated tumor-associated T_reg_ cells (Figure 4D). This collectively formed a *CD4*^+^ *LAIR2*^+^ TILs signature that was prognostically validated across NSCLC microarray and RNA sequencing datasets (Figure 5). It was unclear why LAIR2 was selectively expressed by tumor-associated T_reg_ cells and not other T subsets within TILs. However, given the highly collagenous nature of the TME, autocrine expression of LAIR2 may provide T_reg_ cells with a survival benefit, as high collagen density has been associated with reduced T cell activation and expression of cytotoxicity markers [23,47,48].

Consistent with its adversely prognostic role in adenocarcinoma NSCLC, we found that LAIR2 could act in *trans* to regulate tumor cell adhesion and invasion. Using an in vitro model of tumor migration into type 1 collagen, we observed an increase in tumor cell invasion in the presence of LAIR2 secreting T cells (Figure 3). These results suggested that LAIR2 may have roles beyond alleviating collagen-induced immune inhibition [23,25] and may function to regulate tumor migration through an unknown mechanism that likely involve its function as a collagen receptor (Figure 5C). Though our study did not explore the role that LAIR2 may have in checkpoint blockade immunotherapies, several studies have highlighted a predictive role for tumor-infiltrating T_reg_ cells in response to PD-1/PD-L1 blockade. The presence of PD-1^+^ or PD-L1^+^ T_reg_ cells was predictive of response to checkpoint inhibitors, indicating a dependency on this pathway, to which blockade increased CD8^+^ T cell responses [49,50,51]. Given this observation, it is likely that LAIR2 have an adverse role in immunity induced by checkpoint inhibition. Taken together, our data support a role for LAIR2 expression in tumor-associated Foxp3^+^ T_reg_ cells. LAIR2, which defines a subset of adversely prognostic T_reg_ cells in lung adenocarcinoma, therefore provides a potential target for immunotherapeutic development.

## Figures and Tables

**Table 1:** Univariate and multivariate analysis of *LAIR2* expression.

Parameter	Univariate Analysis			Multivariate Analysis		
	HR	95% CI	*p*-Value	HR	95% CI	*p*-Value
*LAIR2* (high vs. low)	2.08	1.20–3.64	0.0071	1.96	1.05–3.66	0.03
*LAIR1* (high vs. low)	1.62	0.94–2.80	0.083	1.16	0.62–2.15	0.64
Age (older vs. younger)	1.00	0.99–1.1	0.12	1.02	0.99–1.05	0.13
Sex (female vs. male)	0.76	0.44–1.3	0.33	0.75	0.42–1.36	0.35
Smoking (no vs. yes)	0.96	0.62–1.5	0.87	0.99	0.62–1.59	0.97
Stage (Stage II vs. I)	2.4	1.4–4.2	0.002	2.21	1.22–3.98	0.008
Histological Pattern (micropapillary/solid vs. acinar/papillar/lepidic)	0.95	0.63–1.4	0.79	0.92	0.61–1.4	0.71

*p*-value, determined by log rank test.

## Data Availability

Datasets available in publicly accessible repositories as indicated in Section 2.

## Supplementary Materials

The following are available online at https://www.mdpi.com/article/10.3390/cancers14010205/s1, Table S1. Patient demographics, Tables S2 and S3. Gene lists for LAIR2 associated genes and signatures, Figure S1. LAIR1 expression is not prognostic in LUAD, Figure S2. GSEA analysis of indicated gene sets correlating to LAIR2 expression. GSEA analysis shows that LAIR2 expression is correlated to signatures of T cell exhaustion. GSEA plots are presented as described in [35], where the green line represents the enrichment score for the gene set, the black bar-code represents members of the gene set contributing most to the enrichment score and their location along the ranked list, and the gray bar represents the value of the ranking metric. See https://www.gsea-msigdb.org/gsea/doc/GSEAUserGuideFrame.html?Interpreting_GSEA (accessed on 13 December 2021) for detailed description of GSEA analysis and interpretation. Normalized enrichment score (NES), FDR q-value (q) and ranked list method (Metric) are shown, Figure S3. scRNASeq analysis indicates that LAIR2 is pre-dominantly expressed by tumor-associated T_reg_ cells. A, LAIR2, and LAIR1 expressing cells rarely co-localize. Left, t-SNE plot of LUAD TILs showing indicated genes. Right, dot plot of indicated genes gated using (TPM + 1) > 4 as cut-off for expression. B, t-SNE analysis of T_reg_ associated genes. Displayed are t-SNE dimensions 1 and 2, with each data point representing a single cell, colored by the intensity of expression log2(TPM + 1) of indicated gene, (see https://marissafahlberg.com/a-basic-overview-of-using-t-sne-to-analyze-flow-cytometry-data/, accessed on 13 December 2021, for detailed primer), Figure S4. LAIR2 expression correlates with immune subset signatures. A, Left, immune cell estimates were performed on the upper and lower quartiles of LAIR2 expressing patients (UHN181 LUAD) and immune subset enrichment was clustered by high or low LAIR2 expression. Each row represents the normalized enrichment score of immune cell population as estimated by xCell. Each column represents a tumor sample classified as either having high or low LAIR2 expression. Right, immune enrichment score as classified by xCell for patients with high or low LAIR2 expression. Each dot represents a patient, *p*-value was determined by two-tailed, pairwise Mann–Whitney test. B, Spearman rank correlation of immune enrichment score with LAIR2 expression. T central memory (Tcm), T helper 1 (Th1), T effector memory (Tem), natural killer T (NKT), gamma delta T (Tgd), natural killer (NK), plasmacytoid dendritic cells (pDC), T helper 1 (Th2), dendritic cells (DC), conventional dendritic cells (cDC), immature dendritic cells (iDC), and other subset signatures are shown, as described in [37], Figure S5. CD4^+^ LAIR2^+^ gene signature is prognostic in NSCLC validation. Kaplan–Meier plots showing OS of LUAD patients in indicated datasets when normalized to A and C, CD4^+^ T cells or B and D, CD8^+^ T cells. Patients were divided into two risk groups based on the median value of the gene signature risk scores. Number at risk indicates the number of participants who were event free at indicated times.

## Abbreviations

NSCLC: non-small cell lung cancer; LUAD: lung adenocarcinoma; LUSC: lung squamous cell carcinoma; TILs: tumor infiltrating T lymphocytes; TME: tumor microenvironment; scRNASeq: single cell RNASeq; Treg: regulatory T cells.
