# Supplementary material for: Tumor-Associated Regulatory T Cell Expression of LAIR2 Is Prognostic in Lung Adenocarcinoma
Source: Cancers (Basel). 2021 Dec 31;14(1):205. doi: 10.3390/cancers14010205 (PMC8744930; doi:10.3390/cancers14010205)
Supplement: Supplementary file 1 [file cancers-14-00205-s001.zip › LAIR2_MS_Supplementary_Table S1_R1.pdf]

Supplementary Table S1. Clinical pathological markers and association to the LAIR2 expression.

Patient demographic Characteristics		Total (%)	LAIR2 Expression		
			Low (n = 64)	High (n = 64)	p Value
Age	<=65	40 (31.2)	17 (26.6%)	23 (35.9%)	0.25
	>65	88 (68.7)	47 (73.4%)	41 (64.1%)	
Sex	Male	65 (50.7)	29 (45.3%)	36 (56.3%)	0.21
	Female	63 (49.2)	35 (54.7%)	28 (43.7%)	
Smoking status	Never-smoker	23 (17.9)	12 (18.8%)	11 (17.2%)	0.93
	Ever-smoker	92(71.8)	47(73.4%)	45(70.3%)	
	Unknown	13 (10.1)	5 (7.8%)	8 (12.5%)	
Stage	IA	36 (28.1)	20 (31.3%)	16 (25.0%)	0.12
	IB	56 (43.7)	32 (50.0%)	24 (37.5%)	
	IIA	7 (5.4)	2 (3.1%)	5 (7.8%)	
	IIB	29 (22.6)	10 (15.6%)	19 (29.7%)	
Predominant Histological Pattern	Lepidic	6 (4.7)	3 (4.7%)	3 (4.7%)	0.06
	Acinar	29 (22.6)	16 (25.0%)	13 (20.3%)	
	Papillary	32 (25.0)	20 (31.3%)	12 (18.8%)	
	Micropapillary	4 (3.1)	4 (6.3%)	0 (0%)	
	Solid	30 (23.4)	10 (15.6%)	20 (31.3%)	
	Unknown	27 (21.1)	11 (17.2%)	16 (25%)	
