# Supplementary material for: Tumor-Associated Regulatory T Cell Expression of LAIR2 Is Prognostic in Lung Adenocarcinoma
Source: Cancers (Basel). 2021 Dec 31;14(1):205. doi: 10.3390/cancers14010205 (PMC8744930; doi:10.3390/cancers14010205)
Supplement: Supplementary file 1 [file cancers-14-00205-s001.zip › LAIR2_MS_Supplementary_Tables_S2-S3_R1.pdf]

Top500_LAIR2 associated probes/ genes

Affymetrix_ID	Gene Symbol (NetAffy_Mapped)	Pearson R	P-value
207509_s_at	LAIR2	1.00	0.000
211101_x_at	LILRA2	0.58	0.000
208594_x_at	LILRA6 /// LILRB2 /// LILRB3	0.56	0.000
229597_s_at	WDFY4	0.56	0.000
224388_s_at	COL25A1	0.52	0.000
1568949_at	PITPNC1	0.51	0.000
232456_at	C10orf71	0.51	0.000
233467_s_at	TSPAN32	0.50	0.000
206981_at	SCN4A	0.50	0.000
203331_s_at	INPP5D	0.50	0.000
215771_x_at	RET	0.50	0.000
244886_at	LOC389641	0.49	0.000
210324_at	C8G	0.49	0.000
218130_at	C17orf62	0.48	0.000
204413_at	TRAF2	0.48	0.000
217170_at	TRAV8-6	0.47	0.000
234377_at	TRBV7-8	0.47	0.000
221331_x_at	CTLA4	0.47	0.000
217354_s_at	HPS1	0.47	0.000
1560935_s_at	LOC284669	0.47	0.000
210864_x_at	HFE	0.47	0.000
214561_at	LILRP2	0.46	0.000
237024_at	LSMEM2	0.46	0.000
237273_at	KCNU1	0.46	0.000
215843_s_at	TLL2	0.46	0.000
211153_s_at	TNFSF11	0.46	0.000
207890_s_at	MMP25	0.46	0.000
222049_s_at	RBP4	0.46	0.000
206690_at	ASIC2	0.46	0.000
232820_s_at	GTSF1L	0.46	0.000
229502_at	CHDH	0.46	0.000
233523_at	BPIFB4	0.46	0.000
210030_at	---	0.46	0.000
1553007_a_at	TENM1	0.45	0.000
237559_at	GPR55	0.45	0.000
223715_at	BRSK2	0.45	0.000
231203_at	LOC100505478	0.45	0.000
207399_at	BFSP2	0.45	0.000
206916_x_at	TAT	0.45	0.000
206185_at	CRYBB1	0.45	0.000
238397_at	---	0.45	0.000
207421_at	CA5A	0.45	0.000
209474_s_at	ENTPD1	0.45	0.000
219839_x_at	TCL6	0.45	0.000

211269_s_at	IL2RA	0.45	0.000
216234_s_at	PRKACA	0.45	0.000
208224_at	HOXB1	0.45	0.000
1560281_a_at	TMEM95	0.44	0.000
203369_x_at	PDLIM7	0.44	0.000
1553482_at	C15orf32	0.44	0.000
210557_x_at	CSF1	0.44	0.000
239094_at	LOC730961	0.44	0.000
214572_s_at	INSL3	0.44	0.000
233158_at	KRT82	0.44	0.000
209880_s_at	SELPLG	0.44	0.000
204567_s_at	ABCG1	0.44	0.000
214859_at	FSTL4	0.44	0.000
208578_at	SCN10A	0.44	0.000
230481_at	ACY3	0.44	0.000
214847_s_at	GPSM3	0.44	0.000
1555626_a_at	SLAMF1	0.44	0.000
204429_s_at	SLC2A5	0.44	0.000
1557620_a_at	CCDC38	0.44	0.000
238208_at	---	0.44	0.000
213925_at	C1orf95	0.44	0.000
216986_s_at	IRF4	0.44	0.000
208383_s_at	PCK1	0.44	0.000
237257_at	RAB4B	0.43	0.000
233510_s_at	PARVG	0.43	0.000
204890_s_at	LCK	0.43	0.000
209665_at	CYB561D2	0.43	0.000
224054_at	---	0.43	0.000
215540_at	---	0.43	0.000
233823_at	FAM184B	0.43	0.000
207814_at	DEFA6	0.43	0.000
221390_s_at	MTMR8	0.43	0.000
244409_at	CCDC154	0.43	0.000
216668_at	---	0.43	0.000
201401_s_at	ADRBK1	0.43	0.000
1552639_at	KLHDC7B	0.43	0.000
238518_x_at	GLYCTK	0.43	0.000
208198_x_at	KIR2DS1 /// LOC105379650 /// LOC10537	0.43	0.000
231740_at	KCNJ11	0.43	0.000
211249_at	GPR68	0.43	0.000
228572_at	GRB2	0.43	0.000
215925_s_at	CD72	0.43	0.000
242103_at	TMEM86A	0.43	0.000
1561390_at	FAM41AY1 /// FAM41AY2	0.43	0.000
211350_s_at	KIF25-AS1	0.43	0.000
207521_s_at	ATP2A3	0.43	0.000
208064_s_at	ST8SIA3	0.43	0.000

211396_at	FCGR2C	0.42	0.000
224965_at	GNG2	0.42	0.000
202767_at	ACP2	0.42	0.000
222963_s_at	IL1RAPL1	0.42	0.000
1552307_a_at	TTC39C	0.42	0.000
210326_at	AGXT	0.42	0.000
220424_at	NPHS2	0.42	0.000
223899_at	SMIM4	0.42	0.000
208385_at	NR2E3	0.42	0.000
205447_s_at	MAP3K12	0.42	0.000
233426_at	---	0.42	0.000
211389_x_at	KIR3DS1	0.42	0.000
215805_at	---	0.42	0.000
222866_s_at	FLVCR2	0.42	0.000
231671_at	FGA	0.42	0.000
225791_at	UBE2F	0.42	0.000
240434_at	---	0.42	0.000
224557_x_at	P2RX2	0.42	0.000
232178_at	ZNF503	0.42	0.000
204456_s_at	GAS1	0.42	0.000
221360_s_at	GHSR	0.42	0.000
214634_at	HIST1H4I	0.42	0.000
206220_s_at	RASA3	0.42	0.000
1552340_at	SP7	0.42	0.000
216995_x_at	MKRN2	0.42	0.000
231731_at	OTX2	0.42	0.000
232718_at	LINC00589	0.42	0.000
220679_s_at	CDH7	0.42	0.000
210388_at	PLCB2	0.42	0.000
205825_at	PCSK1	0.42	0.000
1554755_a_at	MTUS2	0.42	0.000
210289_at	NAT8	0.42	0.000
1560874_at	CRACR2A	0.42	0.000
210789_x_at	CEACAM3	0.42	0.000
205915_x_at	GRIN1	0.42	0.000
201815_s_at	TBC1D5	0.42	0.000
231457_at	---	0.41	0.000
235597_s_at	RGPD1 /// RGPD2	0.41	0.000
216738_at	---	0.41	0.000
211107_s_at	AURKC	0.41	0.000
242545_at	TTLL11	0.41	0.000
234719_at	LAMA3	0.41	0.000
233072_at	NTNG2	0.41	0.000
202210_x_at	GSK3A	0.41	0.000
220566_at	PIK3R5	0.41	0.000
237188_x_at	SUN5	0.41	0.000
240716_at	TTC23	0.41	0.000

207176_s_at	CD80	0.41	0.000
220727_at	KCNK10	0.41	0.000
211154_at	THPO	0.41	0.000
223615_at	ABI3	0.41	0.000
211900_x_at	CD6	0.41	0.000
217495_x_at	CALCA	0.41	0.000
208266_at	C8orf17	0.41	0.000
1565746_at	IPO5P1	0.41	0.000
1559508_at	---	0.41	0.000
206244_at	CR1	0.41	0.000
207476_at	---	0.41	0.000
1569722_s_at	PROSER2-AS1	0.41	0.000
206923_at	PRKCA	0.41	0.000
210349_at	CAMK4	0.41	0.000
214347_s_at	DDC	0.41	0.000
1555043_at	LHFPL5	0.41	0.000
243387_at	MESP1	0.41	0.000
238177_at	SLC6A19	0.41	0.000
237581_at	---	0.41	0.000
208748_s_at	FLOT1	0.41	0.000
1556662_at	LOC100506142	0.41	0.000
205484_at	SIT1	0.41	0.000
240281_at	---	0.41	0.000
216188_at	MYCNOS	0.41	0.000
221343_at	OR11A1	0.41	0.000
221306_at	GPR27	0.41	0.000
230390_at	LOC101928222	0.41	0.000
221367_at	MOS	0.41	0.000
213985_s_at	TMEM259	0.41	0.000
222784_at	SMOC1	0.41	0.000
217386_at	MRPS11P1	0.41	0.000
203111_s_at	PTK2B	0.41	0.000
222317_at	PDE3B	0.41	0.000
202283_at	SERPINF1	0.41	0.000
227935_s_at	PCGF5	0.41	0.000
1566830_at	LOC440028	0.41	0.000
223745_at	C16orf95	0.41	0.000
202857_at	CNPY2	0.41	0.000
1565735_at	---	0.41	0.000
237536_at	MAPT-AS1	0.41	0.000
1566775_at	DNAH1	0.40	0.000
233215_s_at	ZDHHC21	0.40	0.000
1555611_s_at	MBD1	0.40	0.000
206903_at	EXOG	0.40	0.000
209423_s_at	PHF20	0.40	0.000
1566038_at	DGCR7	0.40	0.000
236061_at	PRDM15	0.40	0.000

1563542_a_at	SCML4	0.40	0.000
238616_at	QDPR	0.40	0.000
223702_x_at	FTCD	0.40	0.000
1564713_a_at	FOXN4	0.40	0.000
227845_s_at	SHD	0.40	0.000
243913_at	---	0.40	0.000
1556265_at	C20orf202	0.40	0.000
229917_at	AGAP2	0.40	0.000
208330_at	ALX4	0.40	0.000
229424_s_at	ARHGAP27	0.40	0.000
214396_s_at	MBD2	0.40	0.000
214022_s_at	IFITM1	0.40	0.000
235491_at	ZBTB10	0.40	0.000
211021_s_at	RGS14	0.40	0.000
216192_at	FABP7	0.40	0.000
1553874_a_at	ZSCAN10	0.40	0.000
240287_at	IRG1	0.40	0.000
241389_at	CHRNB2	0.40	0.000
227519_at	PLAC4	0.40	0.000
1564816_at	C14orf178	0.40	0.000
217057_s_at	GNAS	0.40	0.000
217515_s_at	CACNA1S	0.40	0.000
231028_at	LOC100506082	0.40	0.000
213768_s_at	ASCL1	0.40	0.000
231153_at	C16orf86	0.40	0.000
1562689_at	LOC151484	0.40	0.000
215799_at	LOC101927051	0.40	0.000
221578_at	RASSF4	0.40	0.000
1562841_at	LOC339666	0.40	0.000
242933_at	---	0.40	0.000
206430_at	CDX1	0.40	0.000
217187_at	MUC5AC	0.40	0.000
1566902_at	LOC105378726	0.40	0.000
1555499_a_at	IFNLR1	0.40	0.000
204698_at	ISG20	0.40	0.000
221285_at	ST8SIA2	0.40	0.000
213498_at	CREB3L1	0.40	0.000
219775_s_at	CPLX3	0.40	0.000
206800_at	MTHFR	0.40	0.000
210240_s_at	CDKN2D	0.40	0.000
237098_at	C1orf21	0.40	0.000
1559263_s_at	ZC3H12D	0.40	0.000
243503_at	---	0.40	0.000
234910_at	RASAL2	0.40	0.000
238338_at	DCAF8	0.40	0.000
234819_at	TRAV26-2	0.40	0.000
222727_s_at	SLC8B1	0.40	0.000

1552524_at	ART5	0.40	0.000
221301_at	VWA7	0.40	0.000
206051_at	ELAVL4	0.40	0.000
232642_at	VWA5B2	0.40	0.000
222855_s_at	TRPV2	0.40	0.000
231514_at	C1orf94	0.40	0.000
231741_at	S1PR3	0.40	0.000
239256_at	---	0.40	0.000
1556292_s_at	POM121L12	0.40	0.000
243425_at	LOC101928557	0.40	0.000
211248_s_at	CHRD	0.40	0.000
208280_at	CDRT1	0.40	0.000
210360_s_at	MTSS1	0.40	0.000
233217_at	HSPC102	0.40	0.000
205838_at	GYPA	0.40	0.000
224182_x_at	SEMA6B	0.40	0.000
234714_x_at	ATP2B2	0.40	0.000
1552580_at	TRIML2	0.40	0.000
234271_at	OTOP2	0.40	0.000
206859_s_at	PAEP	0.40	0.000
231589_at	LOC101927685 /// LOC101928917	0.40	0.000
1568248_x_at	SNORA71B	0.39	0.000
229207_x_at	RNF187	0.39	0.000
209994_s_at	ABCB1 /// ABCB4	0.39	0.000
1555015_a_at	ZNF398	0.39	0.000
224422_x_at	PMCHL2	0.39	0.000
209400_at	SLC12A4	0.39	0.000
216955_at	TAF1	0.39	0.000
208189_s_at	MYO7A	0.39	0.000
204951_at	RHOH	0.39	0.000
1557000_at	ESPNL	0.39	0.000
218345_at	TMEM176A	0.39	0.000
206234_s_at	MMP17	0.39	0.000
1568666_at	PLIN5	0.39	0.000
203916_at	NDST2	0.39	0.000
215443_at	TSHR	0.39	0.000
1554383_a_at	TRAM2	0.39	0.000
1555517_at	GABRG3	0.39	0.000
237635_at	LOC100128164	0.39	0.000
209491_s_at	AMPD3	0.39	0.000
210152_at	LILRB4	0.39	0.000
220532_s_at	TMEM176B	0.39	0.000
207311_at	DOC2B	0.39	0.000
1554635_a_at	NPAS3	0.39	0.000
220165_at	INO80D	0.39	0.000
230251_at	LINC00473	0.39	0.000
211253_x_at	PYY	0.39	0.000

1555694_a_at	KCNIP3	0.39	0.000
205718_at	ITGB7	0.39	0.000
233541_at	LIMD1-AS1	0.39	0.000
1555060_a_at	IKZF2	0.39	0.000
218075_at	AAAS	0.39	0.000
237505_at	---	0.39	0.000
1554508_at	PIK3AP1	0.39	0.000
1564709_at	LOC286238	0.39	0.000
216386_at	LOC220077	0.39	0.000
240396_at	---	0.39	0.000
1554201_at	CABP4	0.39	0.000
236689_at	RNF151	0.39	0.000
208494_at	SLC6A7	0.39	0.000
205785_at	---	0.39	0.000
240384_at	---	0.39	0.000
224399_at	PDCD1LG2	0.39	0.000
1552494_at	TAF8	0.39	0.000
1566642_at	---	0.39	0.000
1558450_at	A2M	0.39	0.000
237652_at	IQSEC2	0.39	0.000
216249_at	---	0.39	0.000
206083_at	ADGRB1	0.39	0.000
219896_at	CALY	0.39	0.000
221226_s_at	ASIC4	0.39	0.000
204116_at	IL2RG	0.39	0.000
1563950_at	---	0.39	0.000
221379_at	---	0.39	0.000
227023_at	GLI4 /// ZFP41	0.39	0.000
1569325_at	ARPC5	0.39	0.000
1555339_at	RAP1A	0.39	0.000
204770_at	TAP2	0.39	0.000
210763_x_at	NCR3	0.39	0.000
227182_at	SUSD3	0.39	0.000
208138_at	GAST	0.39	0.000
1570197_at	LOC105372343	0.39	0.000
235139_at	GNGT2	0.39	0.000
229045_at	SNX20	0.39	0.000
222695_s_at	AXIN2	0.39	0.000
1569415_at	---	0.39	0.000
1555628_a_at	HAVCR2	0.39	0.000
221464_at	OR1D2	0.39	0.000
224283_x_at	IL18BP	0.39	0.000
1564251_at	EMID1	0.39	0.000
229496_at	CLP1	0.39	0.000
204425_at	ARHGAP4	0.39	0.000
207868_at	CHRNA2	0.39	0.000
239362_at	NAPA-AS1	0.39	0.000

227549_x_at	ZDHHC24	0.39	0.000
211174_s_at	CCKAR	0.39	0.000
210363_s_at	SCN2B	0.39	0.000
215026_x_at	SCNN1A	0.39	0.000
242720_at	ITIH4	0.39	0.000
217365_at	PRAMEF11	0.39	0.000
241230_at	CA12	0.39	0.000
211123_at	SLC5A5	0.39	0.000
216328_at	SIGLEC8	0.39	0.000
1568683_at	SNAI3-AS1	0.39	0.000
207669_at	KRT83	0.39	0.000
214226_at	PRSS53	0.39	0.000
211886_s_at	TBX5	0.39	0.000
211171_s_at	PDE10A	0.39	0.000
220054_at	IL23A	0.39	0.000
215923_s_at	PSD4	0.39	0.000
207794_at	CCR2	0.39	0.000
211877_s_at	PCDHGA11	0.39	0.000
206508_at	CD70	0.39	0.000
231367_s_at	---	0.39	0.000
1567540_at	---	0.39	0.000
1553803_at	SPATA32	0.39	0.000
207261_at	CNGA3	0.39	0.000
210659_at	CMKLR1	0.39	0.000
1561967_at	LOC105377105	0.39	0.000
211587_x_at	CHRNA3	0.39	0.000
216605_s_at	CEACAM21	0.39	0.000
239874_at	PDE8A	0.39	0.000
206475_x_at	CSH1 /// CSHL1	0.39	0.000
208196_x_at	NFATC1	0.39	0.000
230116_at	KRT8P12	0.39	0.000
224066_s_at	HIPK2	0.39	0.000
244176_at	---	0.39	0.000
206379_at	EYA3	0.39	0.000
1555585_a_at	FAM71B	0.39	0.000
207689_at	TBX10	0.39	0.000
216271_x_at	SYDE1	0.39	0.000
217078_s_at	CD300A	0.38	0.000
201442_s_at	ATP6AP2	0.38	0.000
240070_at	TIGIT	0.38	0.000
234327_at	---	0.38	0.000
1563834_a_at	AKNAD1	0.38	0.000
211688_x_at	KIR3DL1 /// KIR3DL2	0.38	0.000
216537_s_at	SIGLEC7	0.38	0.000
1563878_a_at	LOC338963	0.38	0.000
216963_s_at	GAP43	0.38	0.000
240804_at	---	0.38	0.000

221533_at	FAM162A	0.38	0.000
238226_at	TMEM255B	0.38	0.000
237228_at	ZDHHC1	0.38	0.000
235731_at	AIPL1	0.38	0.000
236554_x_at	TMC8	0.38	0.000
232891_at	SIRPD	0.38	0.000
229209_at	SNHG10	0.38	0.000
1552806_a_at	SIGLEC10	0.38	0.000
208242_at	RAX	0.38	0.000
234883_x_at	TRBV7-3	0.38	0.000
231816_s_at	UBE2Q1	0.38	0.000
212502_at	ADO	0.38	0.000
239416_at	FBXL6	0.38	0.000
206705_at	TULP1	0.38	0.000
208179_x_at	KIR2DL3	0.38	0.000
216083_s_at	NEU3	0.38	0.000
206329_at	EXTL1	0.38	0.000
1553443_at	FER1L6-AS1	0.38	0.000
211598_x_at	VIPR2	0.38	0.000
1552915_at	IFNL2	0.38	0.000
221739_at	MYDGF	0.38	0.000
235816_s_at	RGL4	0.38	0.000
202665_s_at	WIPF1	0.38	0.000
220454_s_at	SEMA6A	0.38	0.000
1554559_at	GPR62	0.38	0.000
230050_at	NACC2	0.38	0.000
204721_s_at	DNAJC6	0.38	0.000
228163_at	ST6GALNAC4	0.38	0.000
210850_s_at	ELK1	0.38	0.000
220091_at	SLC2A6	0.38	0.000
1570470_at	CATSPERB	0.38	0.000
208576_s_at	HIST1H3B	0.38	0.000
205021_s_at	FOXN3	0.38	0.000
236623_at	ATP1A1-AS1	0.38	0.000
225586_at	DPH7	0.38	0.000
230686_s_at	SLC13A3	0.38	0.000
224536_s_at	PCDHGC5	0.38	0.000
1559502_s_at	LRRC25	0.38	0.000
209843_s_at	SOX10	0.38	0.000
231338_at	NUTM1	0.38	0.000
1569909_at	KRT79	0.38	0.000
229079_at	EHMT2	0.38	0.000
232367_x_at	ZNF598	0.38	0.000
224096_at	MIR4755	0.38	0.000
1561273_at	HOXC-AS3	0.38	0.000
227795_at	NDUFV1	0.38	0.000
236702_at	RTFDC1	0.38	0.000

233134_at	RPH3AL	0.38	0.000
243151_at	LOC100506282	0.38	0.000
233619_at	---	0.38	0.000
232310_at	---	0.38	0.000
236894_at	L1TD1	0.38	0.000
207611_at	HIST1H2BL	0.38	0.000
224146_s_at	ABCC11	0.38	0.000
230851_x_at	C16orf13	0.38	0.000
1556426_at	HEXA	0.38	0.000
208060_at	PAX7	0.38	0.000
1557335_at	---	0.38	0.000
216552_x_at	KIR2DS4	0.38	0.000
210569_s_at	SIGLEC9	0.38	0.000
219748_at	TREML2	0.38	0.000
217203_at	---	0.38	0.000
229915_at	FAM26F	0.38	0.000
213767_at	KSR1	0.38	0.000
1553573_s_at	EFNA2	0.38	0.000
219699_at	LGI2	0.38	0.000
220106_at	NPC1L1	0.38	0.000
217041_at	NPTXR	0.38	0.000
229122_x_at	NPRL3	0.38	0.000
224144_at	SPTBN4	0.38	0.000
238404_x_at	SEZ6L2	0.38	0.000
1552549_a_at	BSND	0.38	0.000
1553923_at	SLC22A24	0.38	0.000
217174_s_at	APC2	0.38	0.000
235310_at	GCSAM	0.38	0.000
1557720_s_at	MYO16	0.38	0.000
230969_at	TEX38	0.38	0.000
213297_at	RMND5B	0.38	0.000
224344_at	COX6A1	0.38	0.000
236132_at	TLN1	0.38	0.000
211817_s_at	KCNJ5	0.38	0.000
230382_at	SLC11A1	0.38	0.000
221598_s_at	MED27	0.38	0.000
242267_x_at	LOC105369595	0.38	0.000
214301_s_at	DPYSL4	0.38	0.000
205657_at	HAAO	0.38	0.000
238288_at	---	0.38	0.000
1559533_at	---	0.38	0.000
208426_x_at	KIR2DL4	0.38	0.000
208173_at	IFNB1	0.37	0.000
236003_x_at	OR2I1P	0.37	0.000
237478_at	LOC399900	0.37	0.000
1554442_at	BEST1	0.37	0.000
211521_s_at	CYTH4	0.37	0.000

214228_x_at	TNFRSF4	0.37	0.000
1559136_s_at	LINC00893 /// LINC00894	0.37	0.000
1561136_at	GYPE	0.37	0.000
232419_at	TMEM132A	0.37	0.000
227323_at	COX4I1	0.37	0.000
234828_at	TTC28	0.37	0.000
1552982_a_at	FGF4	0.37	0.000
241012_at	---	0.37	0.000
233410_at	CYP1B1-AS1	0.37	0.000
208262_x_at	MEFV	0.37	0.000
202941_at	NDUFV2	0.37	0.000
239322_at	CEP41	0.37	0.000
243926_at	---	0.37	0.000
211135_x_at	LILRA6 /// LILRB2 /// LILRB3 /// LOC1027	0.37	0.000
1560198_at	LINC00523	0.37	0.000
234361_at	CREB3L3	0.37	0.000
242659_at	---	0.37	0.000
1559712_at	LINC00689	0.37	0.000
236968_at	CCER1	0.37	0.000
237847_at	---	0.37	0.000
238991_at	ASB1	0.37	0.000
232492_at	BVES-AS1	0.37	0.000
227726_at	RNF166	0.37	0.000
237606_at	CD53	0.37	0.000
234886_at	TRBV24-1	0.37	0.000
204943_at	ADAM12	0.37	0.000
233328_x_at	SLC17A9	0.37	0.000
1552436_a_at	CDH23	0.37	0.000
1559478_at	---	0.37	0.000
208431_s_at	TUB	0.37	0.000
210448_s_at	P2RX5	0.37	0.000
1558573_at	MCTS1	0.37	0.000
205346_at	ST3GAL2	0.37	0.000
