# Supplementary figures and images for: Tumor-Associated Regulatory T Cell Expression of LAIR2 Is Prognostic in Lung Adenocarcinoma
Source: Cancers (Basel). 2021 Dec 31;14(1):205. doi: 10.3390/cancers14010205 (PMC8744930; doi:10.3390/cancers14010205)

Figure S1

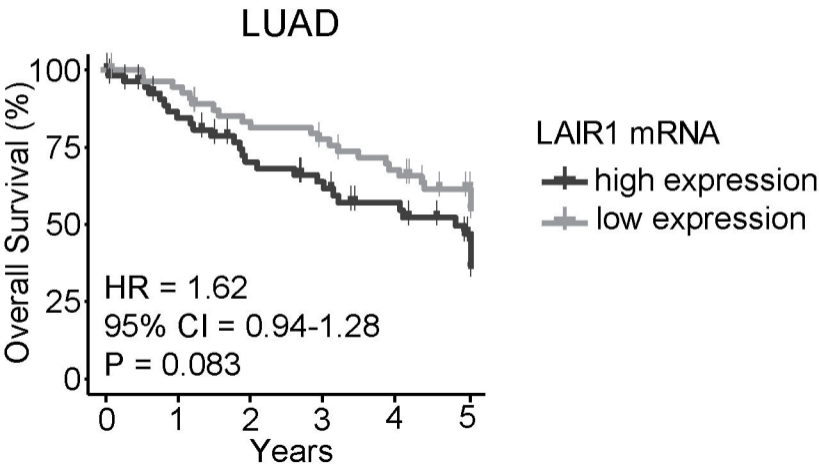

Supplement: Supplementary file 1 [file cancers-14-00205-s001.zip › Supplementary Figure S1_LAIR1_R1.pdf]

Figure S2

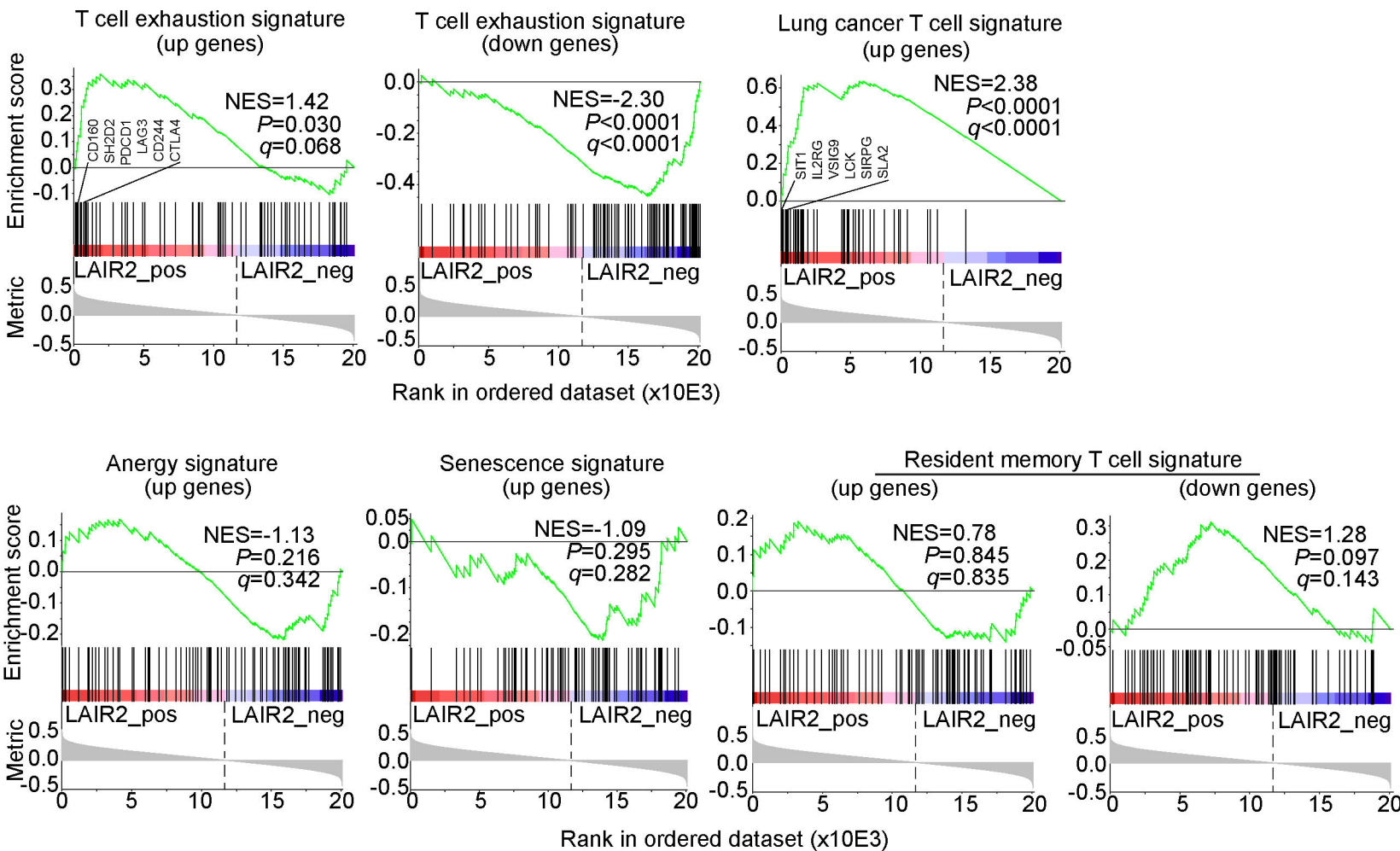

Supplement: Supplementary file 1 [file cancers-14-00205-s001.zip › Supplementary Figure S2_GSEA_R1.pdf]

Figure S3

A

tumor-infiltrating T cells

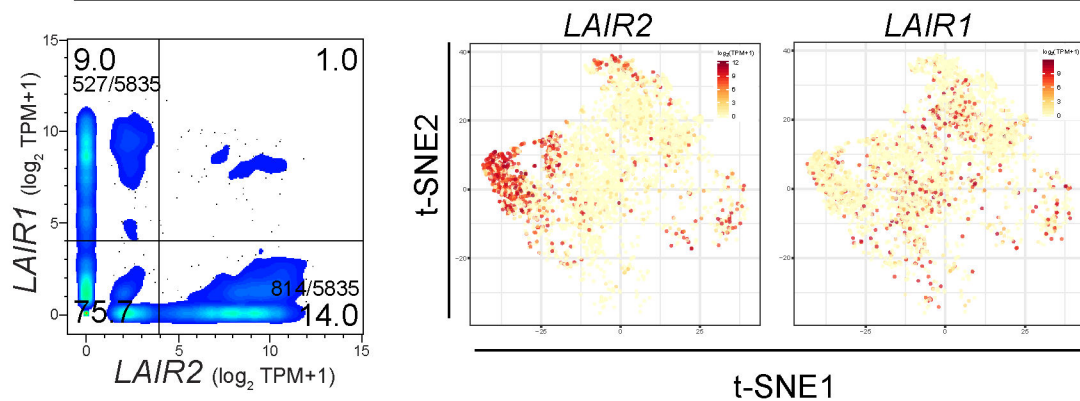

B

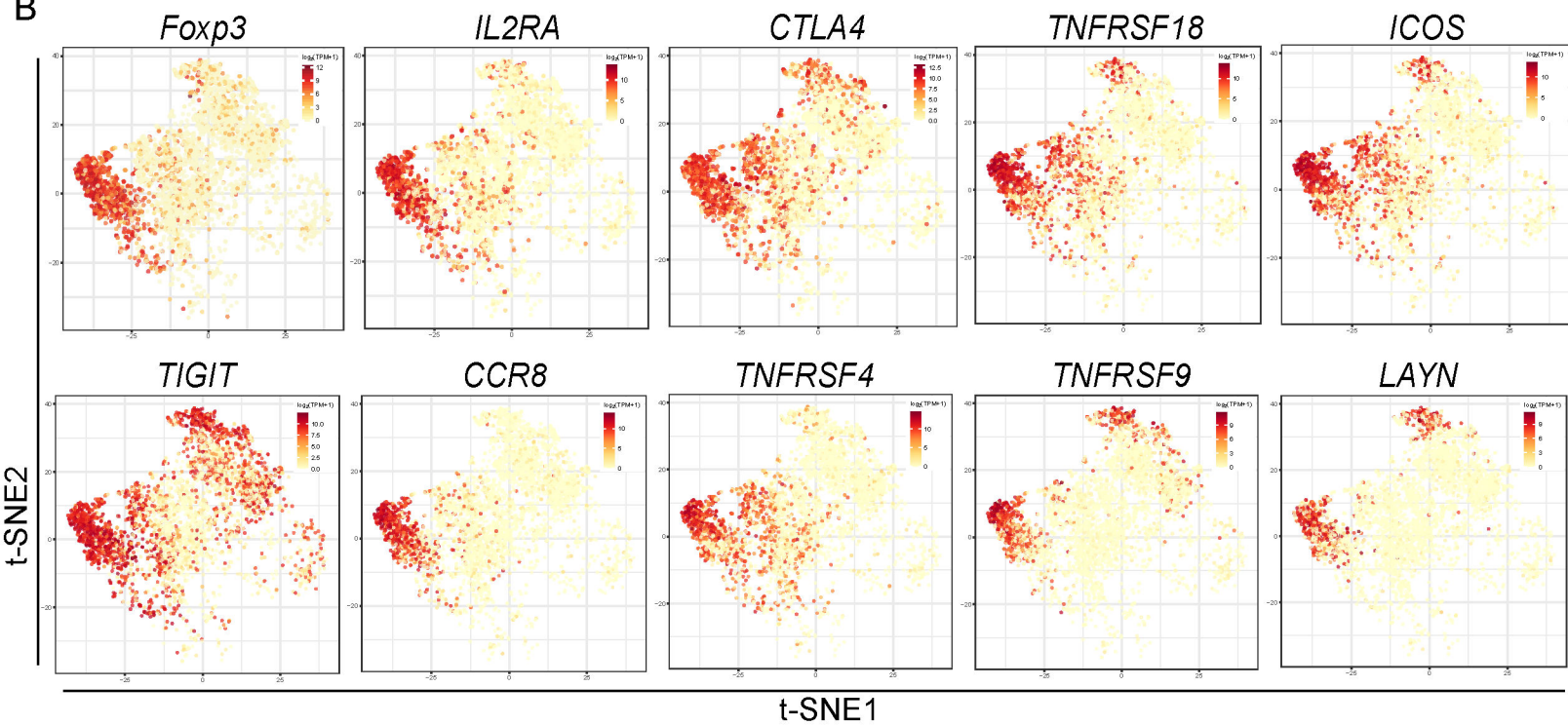

Supplement: Supplementary file 1 [file cancers-14-00205-s001.zip › Supplementary Figure S3_scRNA LAIR1_R1.pdf]

Figure S4

A

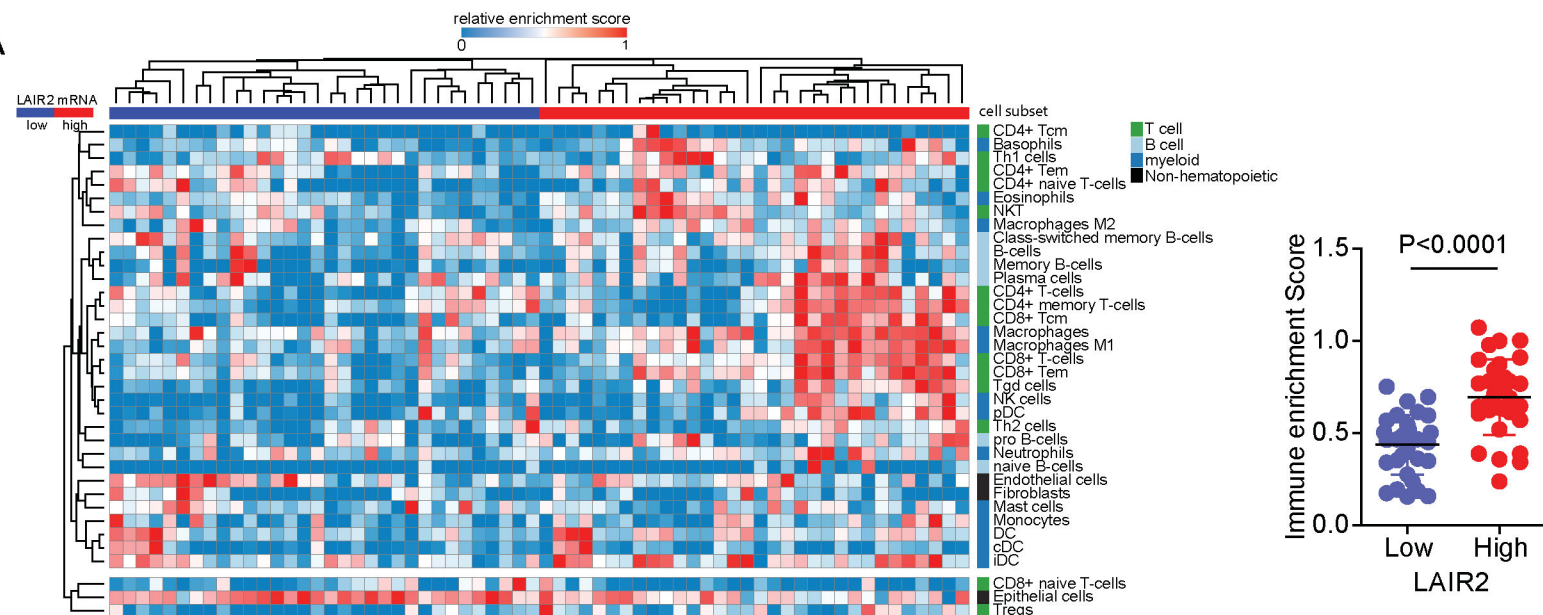

B

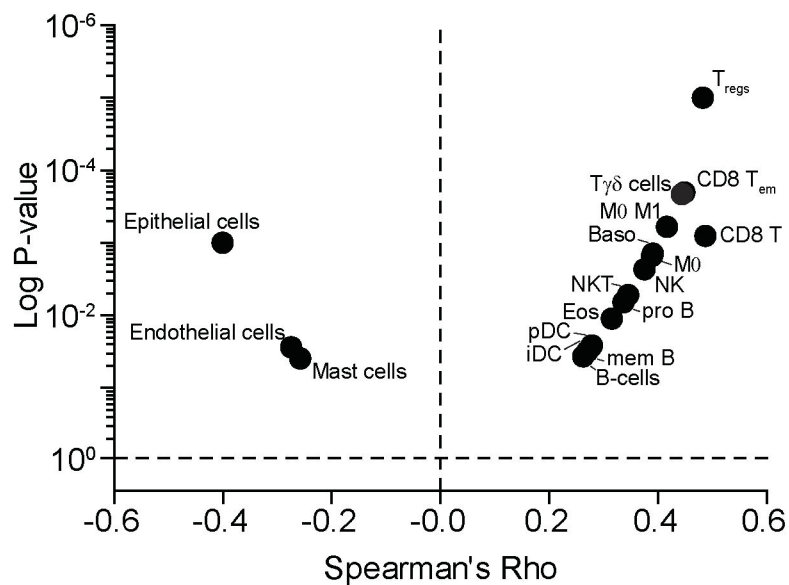

Supplement: Supplementary file 1 [file cancers-14-00205-s001.zip › Supplementary Figure S4_xCell_R1.pdf]

Figure S5

A

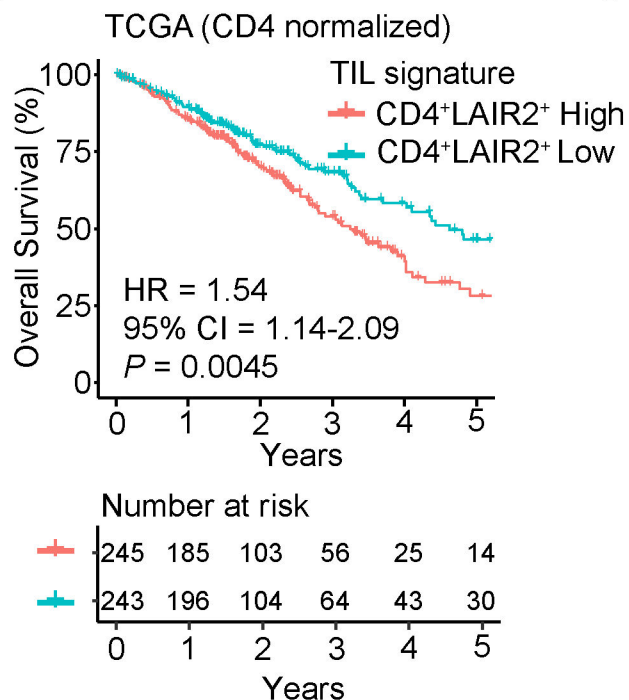

B

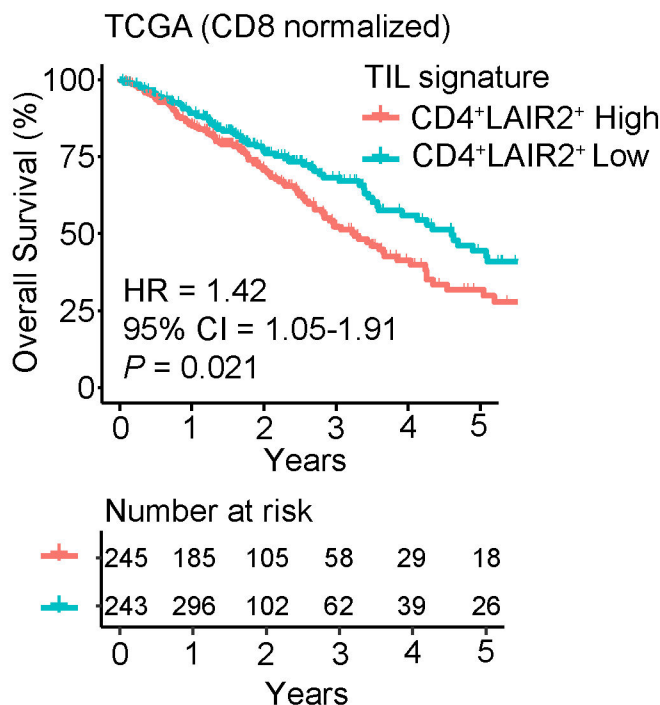

C

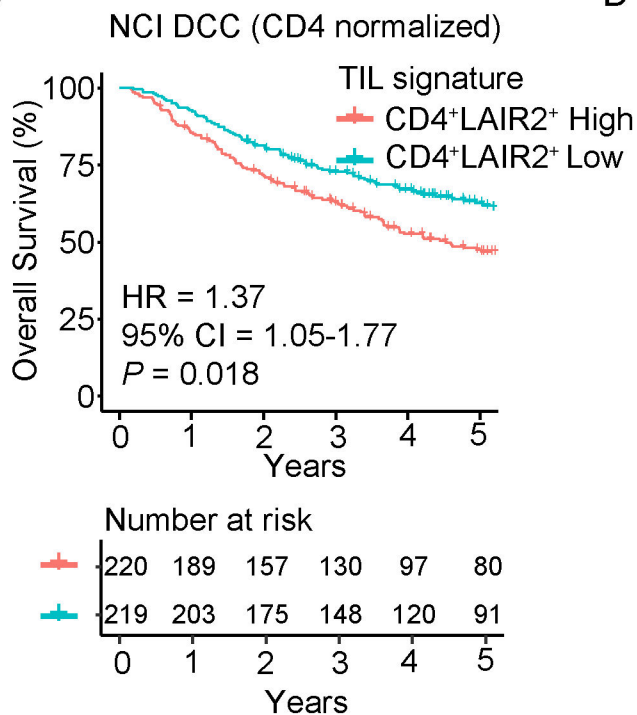

D

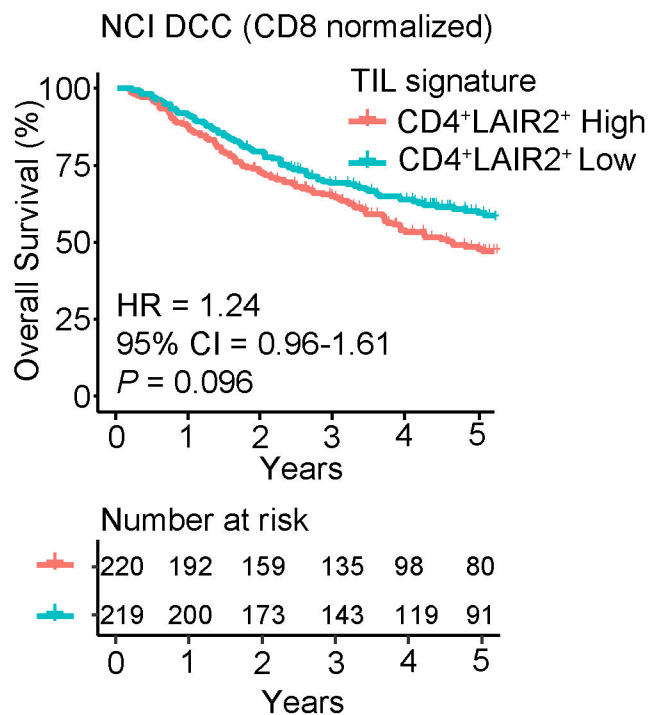

Supplement: Supplementary file 1 [file cancers-14-00205-s001.zip › Supplementary Figure S5_5yr normalized_R1.pdf]
